# Impacts of Denture Retention and Stability on Oral Health-Related Quality of Life, General Health, and Happiness in Elderly Thais

**DOI:** 10.1155/2019/3830267

**Published:** 2019-07-16

**Authors:** Nareudee Limpuangthip, Tewarit Somkotra, Mansuang Arksornnukit

**Affiliations:** ^1^Department of Prosthodontics, Faculty of Dentistry, Chulalongkorn University, Bangkok 10330, Thailand; ^2^Department of Community Dentistry, Faculty of Dentistry, Chulalongkorn University, Bangkok 10330, Thailand

## Abstract

**Purpose:**

This study investigated denture and patient related factors associated with oral health-related quality of life (OHRQoL) of complete denture wearers and their association with general health and happiness.

**Methods:**

This retrospective cohort study comprised 130 participants with complete edentulism, with maxillary and mandibular complete dentures treated at Chulalongkorn University Dental School during 2010-2017. The primary outcome was the presence of overall and domain-specific Oral Impacts on Daily Performances (OIDP). Secondary outcomes were diagnosed and perceived general health, and happiness. Denture retention and stability were classified as acceptable or unacceptable following the CU-modified Kapur criteria. Five esthetic-assessment criteria of the harmonization and proportions between facial and dental anatomical landmarks were measured from patient's photographs. Age, sex, previous complete denture experience, and denture age were recorded. The associations between each variable and oral impacts were analyzed using bivariate logistic regression, and the factors with* p* < 0.25 were further adjusted using multivariable analysis. Associations between oral impact scores and general health and happiness were assessed using Spearman's rank correlation.

**Results:**

The most frequent oral impacts were on physical domain, while social domain was the least affected. Denture retention/stability was significantly associated with both overall and specific domains of oral impact. Happiness was found to be strongly correlated with perceived general health, but marginally with oral impact scores.

**Conclusions:**

Unacceptable complete denture retention and stability are substantial risk factors for impaired OHRQoL in complete edentulism. Maintaining optimal denture retention and stability in denture wearers is essential for good oral health and well-being with the goal of enhancing happiness.

## 1. Introduction

With the increase in life expectancy, tooth loss in elderly individuals has become a global public health concern [1, 2]. People with complete tooth loss, even with prosthetic rehabilitation, may have an impaired oral health-related quality of life (OHRQoL) due to limited masticatory ability and social concerns [3, 4]. Complete denture can assist in improving individual's masticatory ability, solving psychosocial problems, and enhancing oral health [5]. Despite denture use, impaired OHRQoL has been reported by some complete denture wearers [6–8]. However, the cause of impaired OHRQoL has not been well explored in low- to middle-income countries transitioning to an ageing population.

Studies demonstrated an association between patient-self assessments, OHRQoL, patient satisfaction, and denture and/or patient related factors [8, 9]. Denture related factors include denture retention, stability, occlusion, appearance [8–14], and denture age [9]. Patient related factors include age [11], case severity, denture-supporting tissue shape [8, 15], and previous denture experience [13]. However, some studies found no association between patient satisfaction and denture/patient related factors [6, 16]. For elderly Thai complete denture wearers, the underlying determinants of an impaired OHRQoL remain ambiguous. Furthermore, OHRQoL assessed by previous studies provided limited details regarding which domains were frequently affected and by which underlying determinants [4, 7].

Previous studies reported that the most common problem in Thai complete denture wearers was an ill-fitting denture [3, 4, 17], corresponding to the professional terms of denture retention and stability. Recently, the CU-modified criteria have been proposed as an optimal dental-based assessment tool to determine whether a denture needs to be replaced by classifying denture retention and stability into acceptable or unacceptable [3].

Optimal physical and psychosocial well-being are related to an individual's happiness, leading to a longer life expectancy [18]. The Fédération Dentaire Internationale (FDI, World Dental Federation) proposed a new definition of oral health as a person's ability to confidently perform daily activities without any pain or discomfort. Because oral health encompasses physiological, psychological, and social domains that are essential to the quality of life, it is considered as an integral part of health and well-being [19]. However, to our knowledge, the associations between OHRQoL, general health, and happiness in complete denture wearers have not been reported.

Therefore, the objectives of this study were (1) to investigate the underlying denture and patient related factors associated with the OHRQoL of complete denture wearers and determine which domains (physical, psychological, and social) were predominantly affected and (2) to evaluate the associations between OHRQoL and general health and happiness. The research null hypotheses were as follows: (1) there would be no association between OHRQoL and patient and denture related factors, and (2) there would be no associations between OHRQoL, general health, and happiness.

## 2. Materials and Methods

### 2.1. Participants

The participants in this retrospective cohort study comprised 130 removable complete denture wearers treated in the Department of Prosthodontics, Faculty of Dentistry, Chulalongkorn University, Bangkok, Thailand, during 2010-2017. They were selected by stratified random sampling using two subcategories: (1) patient's age and sex; (2) denture age. The inclusion criteria were patients at the Faculty of Dentistry who wore both conventional removable maxillary and mandibular complete dentures for at least 2 years, had no debilitating systemic condition, and could communicate in Thai. The exclusion criteria were patients who refused to provide personal information and/or to have a photograph taken. The data regarding age, sex, denture age, and presence of complete denture experience (yes, no) were obtained from the participant's interview and recorded.

A sample size was calculated from a preliminary study. It was found that the proportions of patients with acceptable and unacceptable denture retention and stability who reported at least one oral impact domain were 0.16 and 0.40, respectively. Based on the research hypothesis, a sample size of 130, including 15% drop-out rate, was required to achieve 90% power with 5% alpha level.

### 2.2. Outcomes

OHRQoL was determined by face-to-face interview using a validated Thai version of the Oral Impacts on Daily Performances (OIDP) index [4, 17].

The OIDP assesses whether a person has difficulty in performing 8 daily activities in 3 domains: physical (eating, speaking, and cleaning); psychological (maintaining usual emotion, smiling/laughing, and sleeping/relaxing); and social (enjoying contacting with people and carrying out major work/social roles) [17]. There are four questions for each daily activity: frequency and severity of the impact, as well as their chief compliant and symptoms caused by their denture. The frequency and severity of each activity or condition-specific (CS) impacts, determined by a five-point ordinal scale (0-5), were multiplied. The full scores of physical, psychological, and social domains were 75, 75, and 50, respectively. The lower score indicated better OHRQoL. The total score of each domain was further classified into presence (score > 0) or absence (score = 0) of oral impact.

Concurrently with OHRQoL, the participants gave their medical history and rated their general health using a five-point ordinal scale: excellent (5), good (4), fair (3), poor (2), and very poor (1). Diagnosed general health was defined as number of underlying diseases as reported by a doctor, e.g., hypertension and diabetes mellitus. Happiness was measured by asking, “taking your own life as a whole, how satisfied are you with your own life?” using a numerical rating scale ranging from 0 (completely dissatisfied) to 10 (completely satisfied), following Yiengprugsawan et al. [20].

### 2.3. Underlying Determinants

Patient and denture related factors were assessed. Considered patient related factors included age (<70 or ≥70 years old), sex (male or female), and presence of complete dentures experience (yes or no). These data were obtained by means of interview. Considered denture related factors included retention/stability (acceptable or unacceptable), esthetic-related criteria (acceptable or unacceptable), and denture age (≤4 years or >4 years). Denture age was obtained from patient's record.

Denture retention and stability were evaluated by a single calibrated examiner (N.L.). Denture retention was defined as the resistance to vertical pulling force, while denture stability was the resistance to horizontal forces [21]. Denture retention and stability were considered as acceptable when the denture resisted displacement from a vertical pull and had slight/no rocking on horizontal movement; otherwise, it was considered as unacceptable. The participants were divided into two groups according to the CU-modified Kapur criteria [3]: (1) acceptable denture retention and stability group and (2) unacceptable either denture retention or stability group or both. One month later, the examiner reevaluated the denture retention and stability in 20 participants. Excellent intraexaminer reliability was achieved as shown by the Kappa score of 0.91-0.99.

Esthetic assessment was performed using the ImageJ program (National Institutes of Health) to measure the facial and dental anatomical landmarks related to the participant's prosthesis from extraoral photographs (Supplementary Materials). Frontal view photographs were taken with a digital camera (Canon EOS 1100D; Canon Inc.; Japan) mounted on a tripod at a constant distance from subjects. The participant's esthetics were assessed using the following criteria [22]: (1) parallelism between the interpupillary line and incisal edge of the maxillary central incisors (parallel, nonparallel), (2) coincidence between the facial and dental midlines (coincidence, noncoincidence), (3) proportion between the maxillary central incisor width and the bizygomatic width = 1:16 (± 10%), (4) proportion between the total maxillary anterior teeth width and the bizygomatic width = 1:3 (± 10%), and (5) proportion of the width of the maxillary central incisor to the lateral incisor to the canine (golden proportion) = 1.618:1:0.618 (± 10%). Each denture esthetic criterion was considered as acceptable (0) when the interpupillary line and incisal edge of the maxillary central incisors were parallel, the facial and dental midlines were coincident, or the three facial-dental proportions met the criteria or within ± 10% error. Otherwise, it was considered as unacceptable (1).

### 2.4. Data Analysis

The percentage distribution of all participants and those who reported oral impacts was calculated according to patient and denture related factors. The main symptoms and chief complaints were identified in the participants reporting oral impacts. Bivariate logistic regression was initially used to determine the association between each underlying factor and presence of each oral impact domain. The factors with a* p*-value less than 0.25 were further adjusted in the multivariable analyses. Considering the oral impacts as continuous variables, the associations between overall and each oral impact domain, as well as general health and happiness scores, were assessed using Spearman rank correlation. The data were analyzed using STATA version 13.0 (StataCorp LP, College Station, TX, USA) at 5% significance level.

## 3. Results

The average age (standard deviation, SD) of the participants was 71.4 (9.3) years old, and 56.2% were female. The participants who had previous complete denture experience was 41.5%. Unacceptable denture retention/stability was identified in 13.8% in the maxillary and 49.2% in mandibular dentures. Approximately 20.0% of the participants possessed nonparallelism or noncoincidence between their dental and facial anatomical landmarks, while the percentages of those with mismatched maxillary anterior teeth width and bizygomatic width, and golden proportion were 89.2% and 100%, respectively (Table 1).

The most frequent oral impacts were on the physical domain (43.0%), especially eating and speaking, while impacts on the social domain were the least frequent (Table 2). The most commonly reported main symptoms were due to functional limitation, pain, and discomfort, predominantly caused by an ill-fitting denture. In contrast, only 5.3% of the participants with smiling/laughing difficulty were dissatisfied with their denture esthetics.

Bivariate and multivariable logistic regression analyses between oral impact domains and underlying factors were demonstrated in Tables 3 and 4. The bivariate analysis indicated that all oral impact domains were more frequently reported by participants with unacceptable either maxillary or mandibular dentures or both (Table 3). In contrast, the participants with noncoincident facial and dental midlines had less frequent oral impacts. However, when all factors were adjusted, there were stronger associations between the oral impacts and unacceptable denture retention/stability (Table 4). Unacceptable denture retention/stability significantly affected the physical and psychological domains. Denture age was not related to the oral impacts.

When the reported oral impacts were considered as continuous variables, there were strong correlations between the overall, physical, and psychological domains of oral impact, while all correlations with the social domain were moderate (Table 5). Happiness strongly correlated with perceived general health, but only marginally with overall and each oral impact domain.

## 4. Discussion

The results showed that OHRQoL was significantly associated with denture quality, but not patient related factors. Also, there were significant associations between OHRQoL, general health, and happiness. Therefore, the null hypotheses were partially rejected. It was found that a major oral impact was on the physical domain, while the social impact was not shown in denture wearers (Table 4), as reported in previous studies of complete denture wearer population [4, 23]. In the present study, the reported oral impacts were common due to functional limitation, pain, and discomfort caused by an ill-fitting denture.

The finding demonstrated the influential impact of unacceptable complete denture retention and stability on impaired OHRQoL of the wearers (Table 4). Interestingly, in this study, other factors such as esthetic-related criteria, denture age, and complete denture experience were not associated with OHRQoL. This result showed the same trend as previous studies which found the associations between retention/stability of maxillary or mandibular denture and OHRQoL, assessed by using Oral Health Impacts Profiles (OHIP) [8, 10, 12]. However, in the present study, the results showed negative impacts of retention and stability of both maxillary and mandibular dentures on general and domain-specific OHRQoL, including physical and psychological impacts (Table 4). Thus, the CU-modified Kapur criteria are suggested as another suitable tool for evaluating complete denture retention and stability and predicting OHRQoL of the wearers.

From the results, it can be implied that esthetics was not a major concern among elderly Thai complete denture wearers. This result was in contrast to the findings of several studies in western countries that demonstrated a major impact of maxillary denture esthetics on the social domain [24, 25]. In addition, we found that nonparallelism between the interpupillary line and the incisal edge of the maxillary central incisor, as well as mismatched facial-dental proportions, did not affect appearance dissatisfaction or psychosocial impacts. Unexpectedly, in the present study, participants with coincident facial and dental midlines reported more frequent oral impacts. It was noted that 65% of them also had unacceptable denture retention/stability, assessed by CU-modified Kapur criteria, which could be a true risk factor of oral impacts. The average value (mean ± SD) among the participants with midline deviation in this study was 1.9 ± 0.8 mm, which might not be noticeable to lay people [26]. Therefore, this might be the reason why an association between esthetic-related criteria and OHRQoL could not be found in the present study.

The data from this study demonstrated the interconnections between oral and general health, quality of life, and happiness (Table 5). The finding revealed that happiness was weakly-to-moderately associated with oral health, but strongly associated with perceived general health, which supported a study on elderly Koreans [27]. This may imply that Thai happiness mainly depended on life circumstances and functional disability [20, 28], rather than oral health. It was also hypothesized that some denture wearers adapted to an ill-fitting denture by avoiding eating hard/tough food and selecting softer items, which might worsen their general health. However, the data showed that up to 60% of the participants who perceived poor/fair general health were wearing a denture with unacceptable retention and/or stability, whereas 65% of whom perceiving good general health were wearing a denture with acceptable retention and stability. The results indicated that oral health may influence happiness indirectly through general health-related quality of life. Therefore, the integration between oral and general health is recommended to help people realize the significance of oral health as a part of their general health and quality of life.

There were limitations to the present study. Information regarding edentulous and denture wearing periods was not included in our analysis because this information was collected from the participants, which might introduce recall bias. In addition, the absolute values of participant's responses such as perceived general health and life happiness responses may differ from other sociocultural contexts. However, it was hypothesized that the relative value of perceived general health and life happiness might reflect the spectrum of these associations. Nevertheless, the participants in this study are likely to be a strong representative of the Thai complete denture wearer population because they were selected using stratified random sampling. All aspects of patient and denture related factors for impaired OHRQoL were also investigated. Moreover, domain-specific and general measures of quality of life, general health, and happiness of complete denture wearers were assessed. At the end of the study, patients who were dissatisfied with the denture due to pain or discomfort, but with acceptable denture retention and stability, underwent a denture adjustment, carried out by postgraduate students under a supervision of the faculty staff. Patients with unacceptable denture retention and stability were recommended to have a new denture fabrication.

## 5. Conclusions

The results suggested that unacceptable complete denture retention and stability are substantial risk factors for impaired OHRQoL in complete edentulism. For complete denture wearers, happiness was strongly associated with perceived general health, but weakly-to-moderately associated with oral health. However, oral health may indirectly impact happiness through general health. Thus, maintaining optimal denture retention and stability in denture wearers is essential for good oral health and well-being with the goal of enhancing happiness.

## Figures and Tables

**Table 1:** Characteristics of the participants and their complete dentures (N = 130).

Characteristics	Distribution, n (%)
Patient related factors	
(1) Age (≥ 70 years)	75 (57.7)
(2) Sex (Female)	73 (56.2)
(3) Presence of complete denture experience	54 (41.5)
Denture related factors	
(1) Unacceptable denture quality: Maxillary denture	18 (13.8)
Mandibular denture	64 (49.2)
(2) Esthetic related criteria:	
Nonparalleled interpupillary line and incisal edge of maxillary central incisor	26 (20.0)
Noncoincidence facial and dental midlines	26 (20.0)
Mismatched proportion; maxillary central incisor: bizygomatic	42 (32.3)
maxillary anterior teeth: bizygomatic	116 (89.2)
maxillary central incisor: lateral incisor: canine	130 (100.0)
(3) Denture age (>4 years)	42 (32.3)

**Table 2:** Distribution of the participants with reported oral impacts according to main symptoms and chief complaints.

Symptoms and Chief complaints	Reported condition-specific impacts; %							
	Physical (42.9)			Psychological (27.8)			Social (7.1)	
	Eating (42.1)	Speaking (23.0)	Cleaning (2.4)	Emotion (19.0)	Smiling/Laughing (15.1)	Sleeping (2.4)	Contacting (7.1)	Working (2.4)
Main symptoms^†^: n%								
Functional limitation	94.3	100.0	0.0	62.5	94.7	0.0	100.0	100.0
Pain/Discomfort	79.3	55.2	66.7	83.3	31.6	100.0	55.6	33.3
Appearance dissatisfaction	0.0	0.0	0.0	0.0	5.3	0.0	0.0	0.0

Main chief complaints^†^: n%								
Ill-fitting denture	81.1	79.3	33.3	75.0	94.7	33.3	88.9	100.0
Chewing pain from denture	50.9	3.4	66.7	20.8	0.0	33.3	0.0	0.0
Bulky denture	22.6	17.2	0.0	33.3	0.0	33.3	11.1	33.3
Bad denture occlusion	22.6	3.4	0.0	0.0	0.0	0.0	0.0	0.0
Food retention	22.6	0.0	0.0	0.0	0.0	0.0	0.0	0.0
Poor esthetics of denture	0.0	0.0	0.0	0.0	5.3	0.0	0.0	0.0

^†^Main symptoms and chief complaints were determined among those with CS-impacts. A person could report more than one symptoms/complaint.

**Table 3:** Odds ratio (95% CI) from bivariate analyses of oral impact domains.

Determinants	Affected domains					
	Physical (42.9%)		Psychological (27.8%)		Social (7.1%)	
	Prevalence (%)	OR (95% CI)	Prevalence (%)	OR (95% CI)	Prevalence (%)	OR (95% CI)
Patient related factors						
(1) Age: < 70 years	33.3	1 (Reference)	23.5	1 (Reference)	5.9	1 (Reference)
≥ 70 years	49.3	1.95 (0.93, 4.07)^†^	30.6	1.44 (0.64, 3.24)	8.0	1.39 (0.33, 5.84)
(2) Sex: Male	44.4	1 (Reference)	33.3	1 (Reference)	5.6	1 (Reference)
Female	41.7	0.89 (0.44, 1.82)	23.6	0.62 (0.28, 1.36)^†^	8.3	1.55 (0.37, 6.48)
(3) Presence of complete denture experience: No	41.7	1 (Reference)	26.4	1 (Reference)	9.7	1 (Reference)
Yes	44.4	1.12 (0.55, 2.28)	29.6	1.17 (0.54, 2.57)	3.7	0.36 (0.07, 1.79)^†^
Denture related factors						
(1) Denture retention and stability:						
Acceptable	6.5	1 (Reference)	4.8	1 (Reference)	0.0	None reported impacts^§^
Unacceptable	75.0	43.5 (13.7, 137.5)*∗∗∗*	47.1	17.5 (5.0, 61.2)*∗∗∗*	13.2	-
(2) Esthetic related criteria:						
Nonparalleled interpupillary line and incisal edge of maxillary central incisor	46.2	1.18 (0.50, 2.82)	26.9	0.95 (0.36, 2.50)	7.7	1.11 (0.22, 5.67)
Noncoincidence facial and dental midline	12.7	0.31 (0.15, 0.67)*∗*	17.3	0.39 (0.16, 0.92)*∗*	1.9	0.16 (0.02, 1.34)^†^
Mismatched proportion: Maxillary central incisor: bizygomatic	47.6	1.34 (0.63, 2.82)	35.7	1.78 (0.79, 3.98)^†^	4.8	0.55 (0.11, 2.77)
Maxillary anterior teeth: bizygomatic	43.1	1.14 (0.30, 4.24)	26.7	0.55 (0.14, 2.07)	6.9	0.67 (0.07, 5.94)
Maxillary central incisor: lateral incisor: canine	42.9	No reference group	27.8	No reference group	7.1	No reference group
(3) Denture age (years): 2 - 4	40.7	1 (Reference)	26.7	1 (Reference)	4.7	1 (Reference)
> 4	47.5	1.32 (0.62, 2.81)	30.0	1.17 (0.51, 2.69)	12.5	2.93 (0.74, 11.6)^†^

Significant association at *∗∗∗p *< 0.001, *∗∗p *< 0.01, *∗p *< 0.05, ^†^*p *< 0.25, ^***§***^no *p*-value available. “None reported impacts” indicated no participants in that subgroup reported oral impacts.

**Table 4:** Adjusted odds ratio (95% CI) from multivariable analyses of oral impact domains.

Determinants	Affected domain; adjusted OR (95% CI)		
	Physical	Psychological	Social
Patient related factors			
(1) Age (≥ 70 years)	2.47 (0.70, 8.64)	1.39 (0.45, 4.33)	0.81 (0.15, 4.38)
(2) Sex (Female)	0.68 (0.20, 2.35)	0.40 (0.13, 1.24)	1.92 (0.37, 10.0)
(3) Presence of complete denture experience	1.45 (0.44, 4.80)	2.07 (0.64, 6.67)	0.36 (0.05, 2.71)
Denture related factors			
(1) Denture retention and stability:			
Acceptable	1 (Reference)	1 (Reference)	None reported impact^§^
Unacceptable	42.8 (13.4, 136.3)*∗∗∗*	17.2 (4.88, 60.8)*∗∗∗*	-
(2) Esthetic related criteria:			
Noncoincidence facial and dental midline	0.15 (0.04, 0.58)*∗∗*	0.23 (0.07, 0.83)*∗*	0.23 (0.02, 2.37)
Unmet proportion of maxillary central incisor: bizygomatic	1.84 (0.53, 6.39)	2.87 (0.91, 9.05)	0.41 (0.06, 2.58)
(3) Denture age (>4 years)	0.64 (0.19, 2.34)	0.62 (0.19, 2.09)	1.93 (0.37, 10.1)

Significant association at *∗∗∗p *< 0.001, *∗∗p *< 0.01, *∗p *< 0.05, ^***§***^no *p*-value available.

“None reported impact” indicated no participants in that subgroup reported oral impacts.

**Table 5:** Spearman correlation coefficient of the scores of oral impact, happiness, and perceived general health.

Outcomes	Oral impact score				Happiness level	Perceived general health
	Overall	Physical	Psychological	Social		
Oral impact score; Physical	0.95*∗∗∗*	1				
Psychological	0.70*∗∗∗*	0.73*∗∗∗*	1			
Social	0.31*∗∗∗*	0.39*∗∗∗*	0.42*∗∗∗*	1		
Happiness level	-0.21*∗*	-0.26*∗∗*	-0.21*∗*	-0.14	1	
Perceived general health	-0.18*∗*	-0.18*∗*	-0.23*∗*	-0.19*∗*	0.51*∗∗∗*	1
Number of underlying diseases	0.07	0.11	-0.01	0.08	-0.13	-0.34*∗∗∗*

Significant association at *∗∗∗p *< 0.001, *∗∗p *< 0.01, *∗p *< 0.05.

## Data Availability

The data used to support the findings of this study are restricted by the Human Research Ethics Committee of the Faculty of Dentistry, Chulalongkorn University, in order to protect patient privacy. However, data are available from the authors upon reasonable request and with permission of the editor.

## Supplementary Materials

Supplementary 1 Figure 1. Parallelism between the interpupillary line and incisal edge of the maxillary central incisors (red lines); coincidence between the facial and dental midlines (black line).

Supplementary 2 Figure 2. The width of total maxillary anterior teeth and maxillary central incisor (black lines) to the bizygomatic width (red line).

## References

[1] Müller F. (2014). Interventions for edentate elders - what is the evidence?. *Gerodontology*.

[2] Müller F., Shimazaki Y., Kahabuka F., Schimmel M. (2017). Oral health for an ageing population: the importance of a natural dentition in older adults. *International Dental Journal*.

[3] Limpuangthip N., Somkotra T., Arksornnukit M. (2018). Modified retention and stability criteria for complete denture wearers: A risk assessment tool for impaired masticatory ability and oral health-related quality of life. *The Journal of Prosthetic Dentistry*.

[4] Srisilapanan P., Sheiham A. (2001). The prevalence of dental impacts on daily performances in older people in Northern Thailand. *Gerodontology*.

[5] Jenei Á., Sándor J., Hegedűs C. (2015). Oral health-related quality of life after prosthetic rehabilitation: a longitudinal study with the OHIP questionnaire. *Health and Quality of Life Outcomes*.

[6] John M. T., Szentpetery A., Steele J. G. (2007). Association between factors related to the time of wearing complete dentures and oral health-related quality of life in patients who maintained a recall. *The International Journal of Prosthodontics*.

[7] Srisilapanan P., Korwanich N., Jienmaneechotchai S. (2016). Estimate of impact on the oral health-related quality of life of older thai people by the provision of dentures through the royal project. *International Journal of Dentistry*.

[8] Yamaga E., Sato Y., Minakuchi S. (2013). A structural equation model relating oral condition, denture quality, chewing ability, satisfaction, and oral health-related quality of life in complete denture wearers. *Journal of Dentistry*.

[9] Cerutti-Kopplin D., Emami E., Hilgert J. B., Hugo F. N., Rivaldo E., Padilha D. M. (2017). Predictors of satisfaction with dentures in a cohort of individuals wearing old dentures: functional quality or patient-reported measures?. *Journal of Prosthodontics*.

[10] Alfadda S. A., Al-Fallaj H. A., Al-Banyan H. A., Al-Kadhi R. M. (2015). A clinical investigation of the relationship between the quality of conventional complete dentures and the patients' quality of life. *The Saudi Dental Journal*.

[11] Celebic A., Knezovic-Zlataric D., Papic M., Carek V., Baucic I., Stipetic J. (2003). Factors related to patient satisfaction with complete denture therapy. *The Journals of Gerontology. Series A, Biological Sciences and Medical Sciences*.

[12] Chen Y., Yang Y., Chen J. (2012). The impact of complete dentures on the oral health-related quality of life among the elderly. *Journal of Dental Sciences*.

[13] van Waas M. A. J. (1990). Determinants of dissatisfaction with dentures: a multiple regression analysis. *The Journal of Prosthetic Dentistry*.

[14] Komagamine Y., Kanazawa M., Kaiba Y., Sato Y., Minakuchi S., Sasaki Y. (2012). Association between self-assessment of complete dentures and oral health-related quality of life. *Journal of Oral Rehabilitation*.

[15] Kurushima Y., Matsuda K., Enoki K., Ikebe K., Maeda Y. (2015). Does case severity make a difference to clinical improvement following complete denture treatment?. *International Journal of Prosthodontics*.

[16] De Lucena S. C., Gomes S. G. F., Da Silva W. J., Del Bel Cury A. A. (2011). Patients' satisfaction and functional assessment of existing complete dentures: Correlation with objective masticatory function. *Journal of Oral Rehabilitation*.

[17] Adulyanon S., Vourapukjaru J., Sheiham A. (1996). Oral impacts affecting daily performance in a low dental disease Thai population. *Community Dentistry and Oral Epidemiology*.

[18] Yiengprugsawan V., Seubsman S.-A., Sleigh A. C. (2014). Unhappiness and mortality: Evidence from a middle-income Southeast Asian setting. *BioPsychoSocial Medicine*.

[19] Glick M., Williams D. M., Kleinman D. V., Vujicic M., Watt R. G., Weyant R. J. (2016). A new definition for oral health developed by the FDI World Dental Federation opens the door to a universal definition of oral health. *International Dental Journal*.

[20] Yiengprugsawan V., Somboonsook B., Seubsman S., Sleigh A. C. (2012). Happiness, mental health, and socio-demographic associations among a national cohort of thai adults. *Journal of Happiness Studies*.

[21] GPT-9 (2017). The glossary of prosthodontic terms: ninth edition. *Journal of Prosthetic Dentistry*.

[22] Kumar M. V., Ahila S. C., Devi S. S. (2011). The science of anterior teeth selection for a completely edentulous patient: A literature review. *Journal of Indian Prosthodontist Society*.

[23] Melas F., Marcenes W., Wright P. S. (2001). Oral Health Impact on Daily Performance in Patients with Implant-Stabilized Overdentures and Patients with Conventional Complete Dentures. *The International Journal of Oral & Maxillofacial Implants*.

[24] Ellis J. S., Pelekis N. D., Thomason J. M. (2007). Conventional rehabilitation of edentulous patients: the impact on oral health-related quality of life and patient satisfaction. *Journal of Prosthodontics*.

[25] Gjengedal H., Berg E., Bøe O. E., Trovik T. A. (2011). Self-reported oral health and denture satisfaction in partially and completely edentulous patients. *International Journal of Prosthodontics*.

[26] Kokich V. O., Kiyak H. A., Shapiro P. A. (1999). Comparing the perception of dentists and lay people to altered dental esthetics. *Journal of Esthetic Dentistry*.

[27] Yoon H., Kim H., Patton L. L., Chun J., Bae K., Lee M. (2013). Happiness, subjective and objective oral health status, and oral health behaviors among Korean elders. *Community Dentistry and Oral Epidemiology*.

[28] Gray R. S., Rukumnuaykit P., Kittisuksathit S., Thongthai V. (2008). Inner happiness among thai elderly. *Journal of Cross-Cultural Gerontology*.

