# Supplementary material for: Impacts of Denture Retention and Stability on Oral Health-Related Quality of Life, General Health, and Happiness in Elderly Thais
Source: Curr Gerontol Geriatr Res. 2019 Jul 16;2019:3830267. doi: 10.1155/2019/3830267 (PMC6662462; doi:10.1155/2019/3830267)
Supplement: Supplementary 1 — Figure 1. Parallelism between the interpupillary line and incisal edge of the maxillary central incisors (red lines); coincidence between the facial and dental midlines (black line). [file 3830267.f1.pdf]

Figure

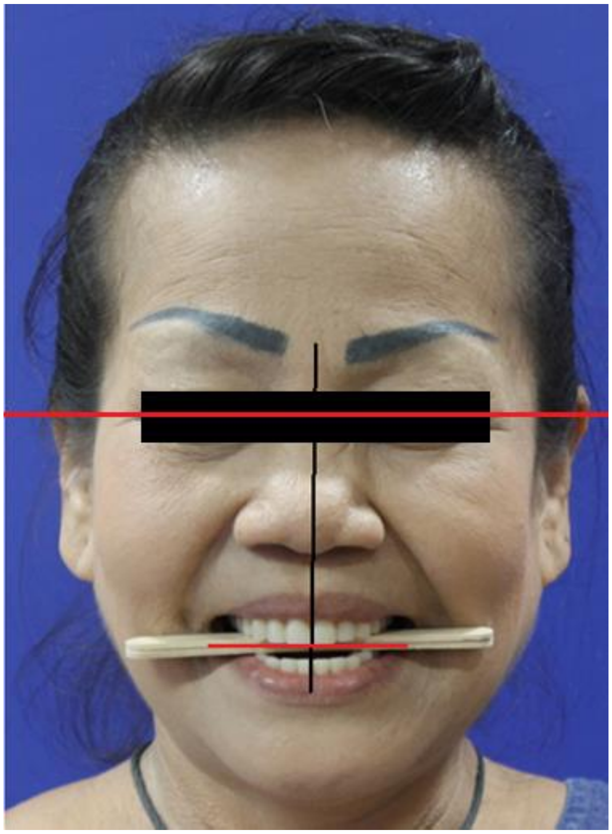

Figure 1: Parallelism between the interpupillary line and incisal edge of the maxillary central incisors (red lines); Coincidence between the facial and dental midlines (black line).
