# Supplementary material for: Impacts of Denture Retention and Stability on Oral Health-Related Quality of Life, General Health, and Happiness in Elderly Thais
Source: Curr Gerontol Geriatr Res. 2019 Jul 16;2019:3830267. doi: 10.1155/2019/3830267 (PMC6662462; doi:10.1155/2019/3830267)
Supplement: Supplementary 2 — Figure 2. The width of total maxillary anterior teeth and maxillary central incisor (black lines) to the bizygomatic width (red line). [file 3830267.f2.pdf]

**Figure**

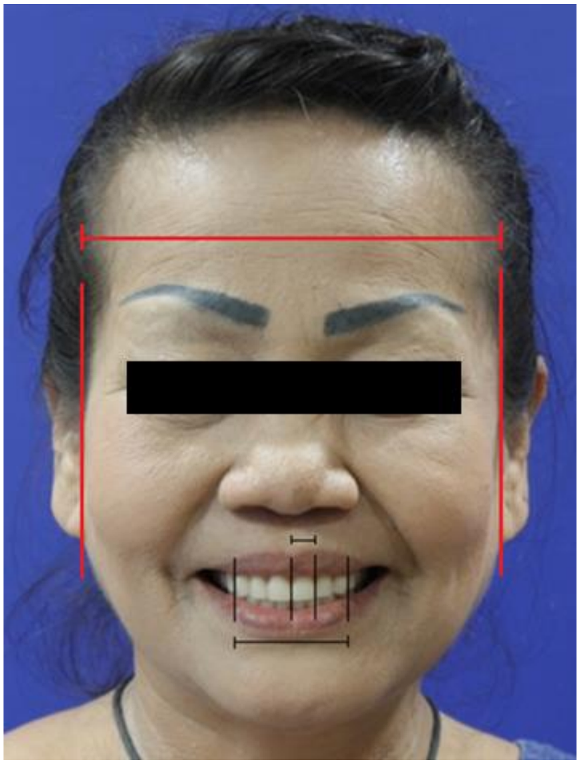

Figure 2: The width of total maxillary anterior teeth and maxillary central incisor (black lines) to the bizygomatic width (red line).
